# Case Report: Telitacicept in the treatment of refractory juvenile idiopathic arthritis: clinical experience from three cases

**DOI:** 10.3389/fped.2026.1846557

**Published:** 2026-07-28

**Authors:** Xiya Luo, Xiwen Luo, Xuemei Tang

**Affiliations:** Department of Rheumatology and Immunology, Children's Hospital of Chongqing Medical University, National Clinical Research Center for Children and Adolescents'Health and Diseases, Ministry of Education Key Laboratory of Child Development and Disorders, Chongqing Key Laboratory of Child Rare Diseases in Infection and Immunity, Chongqing, China

**Keywords:** case report, juvenile idiopathic arthritis, refractory, telitacicept, treatment

## Abstract

**Background:**

Refractory juvenile idiopathic arthritis (JIA) remains difficult to manage, particularly in patients with persistent disease activity despite multiple conventional synthetic disease-modifying antirheumatic drugs and biologic agents. Telitacicept is a dual inhibitor of B-cell activating factor and a proliferation-inducing ligand, but its use in JIA has not been well described.

**Case presentation:**

We report three pediatric patients with refractory JIA treated with telitacicept. Case 1 was a 6-year-old girl with extended oligoarticular JIA who presented with recurrent swelling, pain, and limited motion of multiple joints, and elevated inflammatory markers after previous treatment with methotrexate, leflunomide, adalimumab, and intra-articular glucocorticoids. After switching to telitacicept combined with tofacitinib and methotrexate, joint symptoms and inflammatory markers improved, and an ACR70 response was achieved; clinical improvement was maintained during 12 months of follow-up. Case 2 was a 17-year-old boy with RF-positive polyarticular JIA and long-standing active disease involving the wrists and small joints of the hands. After telitacicept was added to background therapy, joint swelling resolved, inflammatory markers normalized, and ACR90 response was achieved during 9 months of follow-up. Case 3 was an 11-year-old boy with systemic JIA whose systemic manifestations had been controlled but who continued to have predominant polyarticular involvement. After telitacicept initiation, wrist swelling and inflammatory markers improved during the first 2 months, and an early ACR90 response was observed; however, relapse occurred at month 3 with recurrence of systemic symptoms, leading to discontinuation of telitacicept. No serious adverse events were observed. One patient developed a mild upper respiratory tract infection that resolved with symptomatic treatment.

**Conclusion:**

These cases provide preliminary clinical observations on telitacicept use in refractory JIA. BAFF/APRIL inhibition may warrant further investigation in selected patients with refractory JIA, particularly those with persistent articular involvement; however, prospective controlled studies are needed to evaluate efficacy and safety.

## Introduction

Juvenile idiopathic arthritis (JIA) is one of the most common rheumatic diseases in childhood. In recent years, the widespread use of conventional synthetic disease-modifying antirheumatic drugs (csDMARDs) and biologic agents has substantially improved the quality of life in patients with JIA. However, a subset of children continues to exhibit inadequate responses to multiple csDMARDs and biologics (1), remaining in a persistently active disease state and displaying clinical features analogous to difficult-to-treat rheumatoid arthritis (RA) in adults (2). The management of these patients remains challenging, underscoring the need for novel and effective therapeutic strategies. Telitacicept is a novel fusion protein that modulates B-cell function by simultaneously inhibiting B-cell activating factor (BAFF) and a proliferation-inducing ligand (APRIL) (3). On July 16, 2024, telitacicept was officially approved by the National Medical Products Administration of China for the treatment of RA. However, evidence supporting its use in JIA is currently lacking. Against this background, we report three pediatric patients with refractory JIA who were treated with telitacicept, and we provide a preliminary evaluation of its efficacy and safety, aiming to offer clinical insight into its potential application in JIA.

## Case presentation

All three patients met the 2001 ILAR classification criteria for JIA after infections, malignancies, and other rheumatic diseases had been excluded (4). The clinical characteristics of the three patients are shown in Table 1. Refractory JIA was defined as persistent disease activity despite treatment failure with csDMARDs and biologic agents involving three distinct mechanisms of action. Each patient had previously received multiple csDMARDs and biologics, including methotrexate, tumor necrosis factor-α inhibitors, and tocilizumab, but showed inadequate responses. After comprehensive evaluation and detailed discussions with the families, adalimumab was discontinued and subcutaneous telitacicept was initiated (Patient 1 received concomitant treatment with tofacitinib). The off-label use of telitacicept was approved with written informed consent from the patients' guardians. Outcomes were assessed using the American College of Rheumatology Pediatric response criteria (5). The ACR70 and 90 response definitions require at least 70% and 90% improvement, respectively, from baseline in at least three of the six core variables, with no more than one of the remaining variables worsening by more than 30%.

**Table 1:** Clinical features of 3 JIA patients.

Subject	Case 1	Case 2	Case 3
Sex	Female	Male	Male
Diagnosis	Extended oligoarthritis	Polyarthritis (Rheumatoid Factor Positive)	Systemic Arthritis
Age at JIA diagnosis	2 years 7 months	4 years 6 months	9 years 2 months
Treatments prior to telitacicept	Methotrexate, Leflunomide, Adalimumab	Prednisone, Methotrexate, Hydroxychloroquine, Thalidomide, Etanercept, Adalimumab, Tofacitinib	Prednisone, Methotrexate, Adalimumab, Tofacitinib
Active joints at baseline	Left knee, Right ankle, Left PIP3, Left MCP3, Right PIP2	Bilateral wrists	Bilateral wrists
Time from diagnosis to telitacicept (months)	44	160	23
Duration of treatment with telitacicept (months)	12	9	3
ACR response	ACR70	ACR90	ACR90 (2 m)
Relapse	No	No	Yes

### Case 1

**Figure 1:**
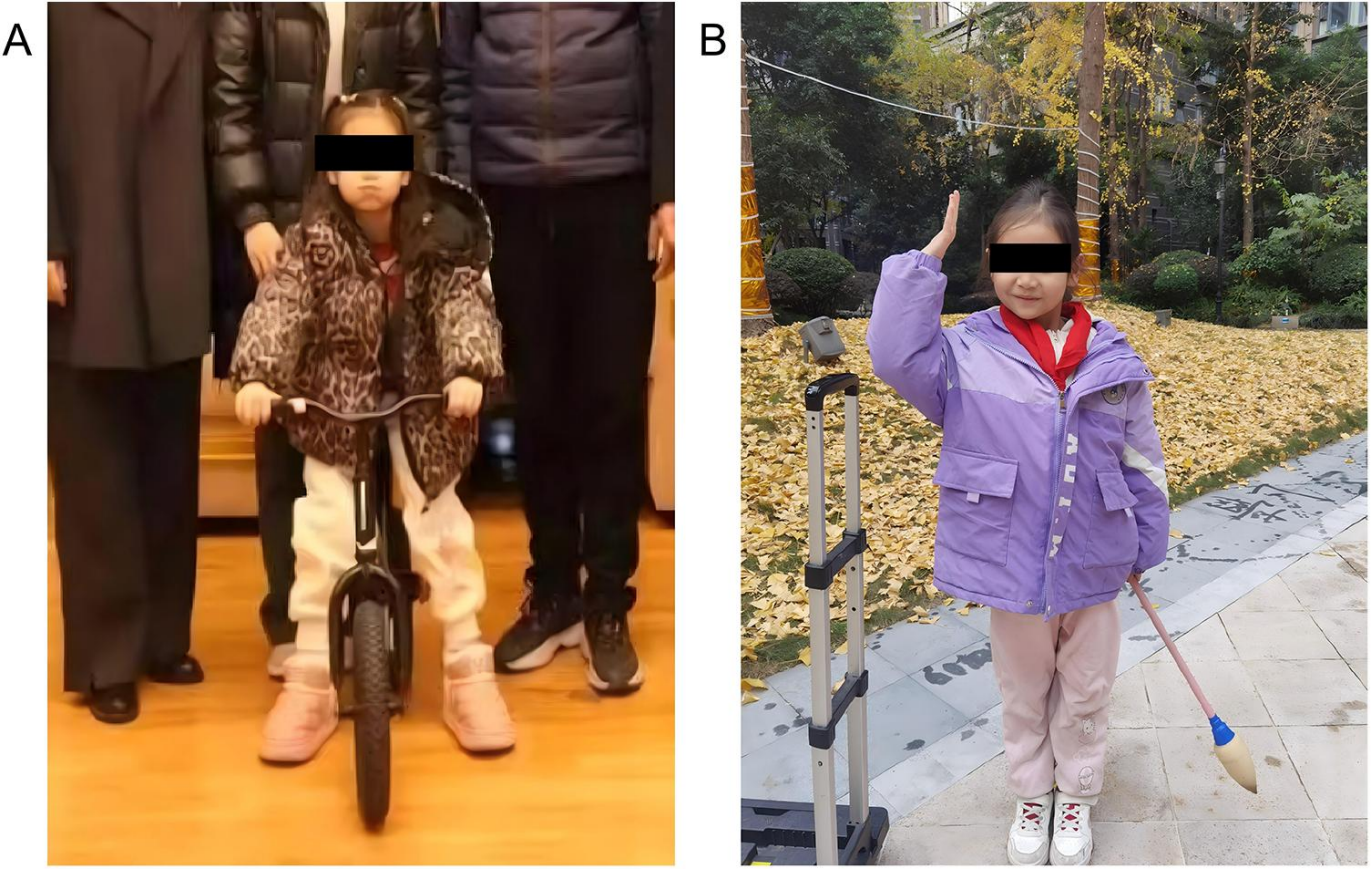
Functional status of case 1 before and after telitacicept treatment. **(A)** Before treatment, case 1 required a mobility aid for ambulation; **(B)** After 12 months of treatment, case 1 was able to walk independently without assistance.

**Figure 2:**
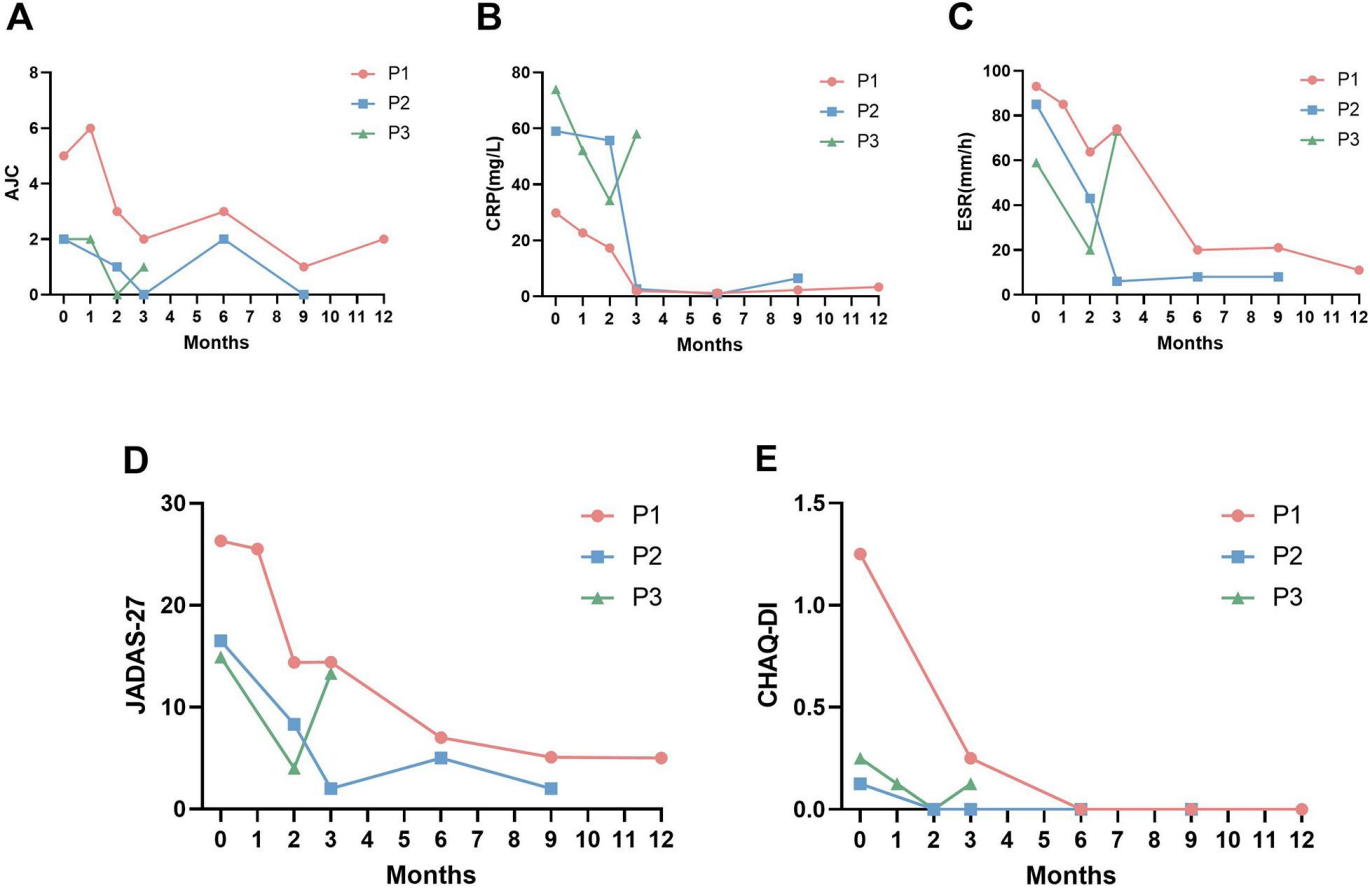
Changes in clinical indicators before and after telitacicept treatment. Telitacicept was initiated at month 0. P1, P2 and P3 were followed for 12, 9, and 3 months, respectively. **(A)** changes in AJC; **(B)** changes in CRP; **(C)** changes in ESR; **(D)** changes in JADAS-27; **(E)** changes in CHAQ-DI. AJC, active joint count; CRP, C-reactive protein; ESR, erythrocyte sedimentation rate; JADAS-27, Juvenile Arthritis Disease Activity Score 27 joints; CHAQ-DI, Childhood Health Assessment Questionnaire - Disability Index.

A 6-year-3-month-old girl had been diagnosed with oligoarticular JIA at another hospital 3 years earlier. She initially presented with swelling, pain, and limited motion of the left knee, with no other joint involvement during the first 6 months. Methotrexate was initiated, but she experienced recurrent flares. Additional therapies, including leflunomide, adalimumab, and intra-articular triamcinolone injections, were subsequently introduced, but with inadequate response. Two weeks before admission, she developed recurrent joint swelling and pain and was referred to our center. On examination, her left knee exhibited swelling, tenderness, limited range of motion, and increased local temperature. Swelling and tenderness were also noted in the right ankle, the metacarpophalangeal and proximal interphalangeal joints of the left middle finger, and the interphalangeal joint of the right index finger. Laboratory tests showed active inflammation, with a C-reactive protein (CRP) level of 29.83 mg/L and an erythrocyte sedimentation rate (ESR) of 93 mm/h. Antinuclear antibodies were positive with a homogeneous pattern at a titer of 1:320. Anti-adalimumab antibodies were elevated at 356 ng/mL (reference <30 ng/mL). Based on her clinical history and physical findings, she was diagnosed with extended oligoarticular JIA. After comprehensive assessment, adalimumab was discontinued, and therapy was switched to telitacicept (80 mg every 10 days) combined with tofacitinib (5 mg in the morning and 2 mg in the evening) and methotrexate, which was continued at the same dose. Because her body weight was low (17.5 kg), telitacicept was administered every 10 days. After 6 months of treatment, joint swelling was markedly reduced, inflammatory markers normalized, and ANA converted to negative. Both the Juvenile Arthritis Disease Activity Score 27 joints (JADAS-27) and Childhood Health Assessment Questionnaire - Disability Index scores (CHAQ-DI) showed significant improvement compared with baseline, and the patient achieved an ACR70 response. The dosing interval of telitacicept was subsequently extended to once every 2 weeks. At 12 months of follow-up, the patient maintained sustained clinical improvement (Figures 1, 2). During treatment, she had one mild upper respiratory tract infection, which was considered possibly related to telitacicept and was classified as mild. Her symptoms resolved after oral symptomatic treatment.

### Case 2

A 17-year-9-month-old boy had been diagnosed with polyarticular juvenile idiopathic arthritis (pJIA, RF-positive) at our center 12 years earlier, initially presenting with recurrent joint swelling and pain. During the first 6 months of disease, more than five joints were involved, and rheumatoid factor was positive on two occasions, supporting the diagnosis. Throughout the disease course, he experienced persistently active systemic inflammation, and his joint symptoms progressively worsened. Whole-genome sequencing did not identify any pathogenic variants. Previous treatments included glucocorticoids, methotrexate, hydroxychloroquine, thalidomide, etanercept, adalimumab, and tofacitinib. On admission, physical examination revealed swelling and tenderness of both wrists, limited range of motion in the metacarpophalangeal and interphalangeal joints of both hands, and an abnormal gait with limping. Laboratory tests showed elevated CRP (59 mg/L) and ESR (85 mm/h). Rheumatoid factor and anti-cyclic citrullinated peptide antibodies were both positive. Following a comprehensive assessment, adalimumab was discontinued, and treatment was switched to telitacicept (160 mg once weekly) in combination with methotrexate, thalidomide, and tofacitinib at the same doses. After 3 months of treatment, joint symptoms improved markedly and inflammatory markers normalized. Anti-cyclic citrullinated peptide antibodies became negative at 6 months. By 9 months, joint swelling had completely resolved, inflammatory markers remained within the normal range, and the patient achieved an ACR90 response. JADAS-27 and CHAQ-DI scores decreased from baseline (Figure 2).

### Case 3

**Figure 3:**
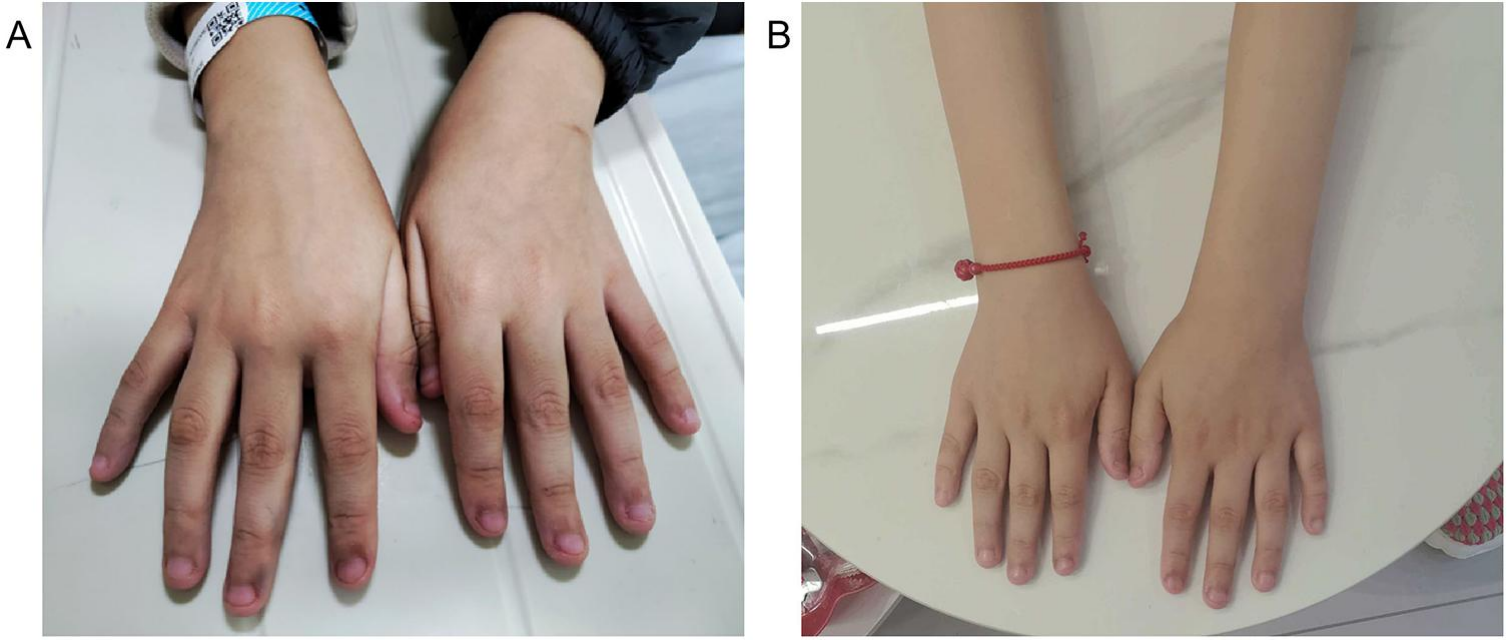
Wrist joint status of case 3 before and after telitacicept treatment. **(A)** Before treatment, case 3 presented with bilateral wrist joint swelling; **(B)** After 2 months of treatment, swelling of both wrists resolved.

An 11-year-1-month-old boy had been diagnosed with systemic JIA (sJIA) at our center 1 year earlier, after presenting with fever, arthritis, and lymphadenitis. He was treated with glucocorticoids plus methotrexate. During the disease course, he experienced recurrent systemic symptoms accompanied by polyarthritis. Sequential therapies, including tocilizumab, adalimumab, and tofacitinib, were administered. Although systemic manifestations were controlled, he continued to have recurrent polyarticular swelling, pain, and limited joint mobility. On admission, physical examination revealed swelling and limited range of motion in both wrists, as well as restricted neck extension. Laboratory tests showed elevated inflammatory markers, with CRP at 73.87 mg/L and ESR at 59 mm/h. Given that no systemic symptoms had occurred for 5 consecutive months and that polyarticular involvement had become the predominant clinical feature, adalimumab was discontinued after comprehensive evaluation. Treatment was switched to telitacicept (80 mg once weekly) in combination with glucocorticoids, methotrexate, and tofacitinib at the same doses. After 2 months, swelling of both wrists resolved (Figure 3), CRP decreased to 34.32 mg/L, ESR returned to the normal range (Figure 2), and an early ACR90 response was observed. However, at month 3, joint symptoms and inflammatory markers worsened, followed by the recurrence of systemic manifestations, including fever. Telitacicept was subsequently discontinued, and therapy was switched to the interleukin (IL) -1 inhibitor firsekibart, which led to improvement in systemic inflammation after 3 months of treatment.

In this case series, all three patients showed varying degrees of clinical and laboratory improvement following treatment with telitacicept. Case 1 achieved an ACR70 response, while Case 2 achieved an ACR90 response, suggesting that clinical improvement may be observed in some patients with JIA characterized predominantly by polyarticular involvement. Case 3 also achieved an ACR90 response in the early phase of treatment; however, disease relapse subsequently occurred, indicating that the therapeutic effect of telitacicept in this subgroup remains uncertain. From the patients' perspective, subcutaneous administration was considered more convenient than intravenous infusion. Patients 1 and 2 reported satisfaction with the treatment response, accompanied by reductions in patient/parent global assessment scores (Supplementary Table S1).

Safety was monitored throughout follow-up. At each visit, patients were assessed for symptoms suggestive of infection and underwent physical examination. Laboratory monitoring included complete blood counts, liver and renal function tests, inflammatory markers, and serum immunoglobulin levels when financially feasible. No serious adverse events occurred. One patient developed a mild upper respiratory tract infection, which did not lead to permanent discontinuation of telitacicept.

## Discussion

This study describes three pediatric patients with refractory JIA. All patients had a long disease duration and had previously received multiple csDMARDs and biologic agents, yet treatment responses were unsatisfactory, with persistently moderate-to-high disease activity. After telitacicept initiation, all three showed varying degrees of clinical improvement, including reductions in joint swelling and pain, lower inflammatory markers, and improved disease activity scores.

Current JIA treatments include nonsteroidal anti-inflammatory drugs, glucocorticoids, csDMARDs, biologic agents, and targeted small-molecule therapies such as Janus kinase (JAK) inhibitors. Among biologic therapies, tumor necrosis factor inhibitors and IL-1/6 inhibitors are the most commonly used. These therapies have substantially improved overall prognosis, but some patients remain persistently active despite multiple lines of therapy, creating ongoing management challenges.

In adult RA, difficult-to-treat disease is generally defined as persistent disease activity despite prior treatment with at least two biologic disease-modifying antirheumatic drugs or targeted synthetic disease-modifying antirheumatic drugs with different mechanisms of action (2). In pediatric JIA, refractory sJIA is commonly described as an inadequate response to IL-1 and/or IL-6 inhibitors, or the need for prolonged glucocorticoid therapy beyond 6 months with ongoing systemic and/or articular manifestations (6). However, no universally accepted definition exists for refractory disease in other JIA subtypes. A systematic review by Chaplin et al. highlighted that refractory pJIA is characterized by resistance to multiple therapies with distinct mechanisms of action, clinically manifesting as persistent symptoms and disease activity, often accompanied by other contributing factors (7). Although the definition of refractory JIA has not been fully standardized, there is general agreement that it centers on two key features: persistent disease activity and failure of multiple therapeutic agents.

Against this background, alternative therapeutic strategies for refractory JIA are being actively explored. A single-center study from Bangladesh defined refractory polyarticular-course JIA as failure to respond to methotrexate and adjunctive therapies within 6 months, and confirmed the efficacy of tofacitinib in this population (8). A French single-center study further evaluated another JAK inhibitor, baricitinib, in refractory JIA. Among seven patients with different JIA subtypes, four achieved complete or partial remission, suggesting potential benefits of JAK inhibition in this setting (9). Hematopoietic stem cell transplantation has also been used as salvage therapy for severe disease refractory to conventional treatment. An international multicenter cohort study reported that some patients with RF-negative pJIA achieved sustained remission following hematopoietic stem cell transplantation; however, this approach was associated with significant risks, including infection-related mortality and long-term relapse (10), underscoring the need for careful risk–benefit assessment. Regarding B-cell–targeted therapy, an Italian single-center study including 37 patients with refractory JIA reported an overall response rate of 73% at 6 months following treatment with rituximab, with nearly half of the patients achieving remission. Nevertheless, a proportion of patients discontinued therapy due to adverse events, and the incidence of infections was relatively high, with some cases requiring hospitalization (11). Overall, although these therapeutic approaches expand the treatment options for refractory JIA, responses vary and safety concerns remain, supporting the need to explore novel agents with distinct mechanisms of action.

Current guidelines generally recommend switching to another biologic agent, such as tocilizumab or abatacept, for patients with pJIA who have an inadequate response to TNF inhibitors (12); IL-1 and IL-6 inhibitors are recommended as first-line biologic options for sJIA (13). In China, however, biologic options within the medical insurance reimbursement system remain limited for patients with JIA who fail TNF-α monoclonal antibody therapy. Baricitinib has only recently been approved for JIA in China. Tofacitinib entered the Chinese market earlier and is therefore more familiar and accessible to physicians. Tocilizumab, infliximab, and rituximab require regular hospital-based intravenous infusions, which may reduce acceptance among some patients and families. Abatacept and anakinra remain largely unavailable in China. In this context, telitacicept was selected for these three patients with refractory JIA.

Pathogenesis studies suggest that innate immunity predominates in sJIA, whereas adaptive immune responses are major drivers in non-sJIA (14). Nevertheless, sJIA may represent a dynamic spectrum that can evolve from autoinflammation toward autoimmune features (15). B cells may play an important role by producing autoantibodies, presenting antigens, activating T cells, and releasing proinflammatory cytokines (16). Although sJIA is considered an autoinflammatory disease, some patients may develop a chronic articular phenotype over time. In this stage, the disease appears to be more driven by B- or T-cell–mediated adaptive immune dysregulation, suggesting a shift toward autoimmune features in the later course of sJIA (17). BAFF and APRIL bind to their receptors to promote B-cell differentiation, proliferation, immunoglobulin production, and upregulation of effector molecule expression (18). Elevated serum levels of BAFF and APRIL have been reported in patients with JIA, including oJIA, pJIA, and sJIA, as well as in adults with RA (19, 20). Therefore, targeting the BAFF/APRIL pathway to modulate B-cell function may offer potential therapeutic benefit in controlling the chronic inflammatory response in certain forms of JIA. Telitacicept inhibits the activation of B cells and plasma cells by simultaneously blocking BAFF and APRIL (3). High-quality evidence has demonstrated its significant efficacy in patients with SLE (21), further supporting its clinical value as a B-cell–targeted therapy for autoimmune diseases. We therefore included one patient each with oJIA, pJIA, and sJIA to explore telitacicept in refractory JIA. Clinical improvement was observed in the patients with oJIA and pJIA. In the patient with sJIA, articular manifestations improved initially, but relapse occurred after 3 months. Because systemic manifestations of sJIA are more closely associated with innate immune activation (15), telitacicept may be less suitable for controlling systemic disease features.

Previous studies have provided preliminary evidence for telitacicept in RA. In a small cohort study, 9 of 10 patients treated with telitacicept achieved a moderate or good EULAR response at 3 months with dosing regimens of 180 mg once or twice weekly (22), suggesting favorable efficacy. More robust evidence comes from a randomized, double-blind, placebo-controlled phase III trial, which demonstrated that telitacicept is both effective and well tolerated in patients with moderate-to-severe RA who had an inadequate response to methotrexate. Compared with placebo, telitacicept significantly increased ACR20 and ACR50 response rates, reduced disease activity, and slowed radiographic progression (23). In addition, recent case reports suggest that telitacicept may offer promising therapeutic potential in patients with RA complicated by myasthenia gravis or presenting with overlap syndromes (24, 25). Taken together, we anticipate that telitacicept may provide therapeutic benefits in the treatment of JIA.

Although clinical benefit was observed, the improvement in disease activity in our patients appeared to occur gradually over several months. Unlike cytokine inhibitors that directly block rapidly acting inflammatory pathways, telitacicept modulates B-cell survival and differentiation through inhibition of the BAFF/APRIL pathway (3), and its clinical effects may therefore take time to become fully apparent. This interpretation is also consistent with previous studies of B-cell–targeted therapies in RA, including telitacicept and rituximab, in which meaningful clinical improvement often emerged after several months of treatment (23, 26). However, given the very small sample size of the present case series, this observation should be interpreted with caution.

In terms of safety, telitacicept is generally well tolerated. In a 24-week clinical trials of RA, the incidences of adverse events, serious adverse events, treatment-related adverse events leading to discontinuation, and infections were comparable to those observed in the placebo group, with no deaths reported (23). Evidence regarding its safety in pediatric populations is gradually accumulating. In a multicenter pediatric SLE study conducted in China with 5–26 weeks of follow-up, all adverse events were mild to moderate and resolved with symptomatic management; no serious adverse events, deaths, or treatment discontinuations due to adverse events were observed. Although decreases in immunoglobulin levels were noted in some patients, clinically significant hypogammaglobulinemia was uncommon (27). Compared with B cell–depleting therapies such as rituximab, telitacicept may therefore have a more favorable safety profile. Furthermore, a 12-month multicenter pediatric SLE study led by our center support these observations, showing low rates of serious infections in both the telitacicept and belimumab groups, with no significant difference between the two (28). In the present study, only one case of mild upper respiratory tract infection was observed during 3–12 months of follow-up. Overall, the available evidence suggests that telitacicept has an acceptable and manageable safety profile in both adult and pediatric immune-mediated diseases, providing important support for its potential use in refractory JIA.

This study has several limitations. First, the small sample size limits generalizability. Second, the study lacked a control group. Finally, because all three patients had refractory disease with substantial ongoing disease activity, concomitant immunosuppressive therapies were used during treatment, which further limits the ability to attribute the observed clinical improvements solely to telitacicept.

In conclusion, this study provides preliminary clinical observations on telitacicept in three patients with refractory JIA. The cases included oJIA, pJIA, and sJIA, offering early observations that may support further exploration of B-cell-targeted strategies across different refractory JIA phenotypes. To further evaluate its clinical utility, our center has initiated a multicenter, single-arm study investigating telitacicept in refractory JIA (MR-50-25-078536). Future prospective, multicenter, randomized controlled trials are warranted to provide more robust evidence regarding its efficacy and safety in this population.

## Data Availability

The original contributions presented in the study are included in the article/Supplementary Material, further inquiries can be directed to the corresponding author.
