# Supplementary material for: Case Report: Telitacicept in the treatment of refractory juvenile idiopathic arthritis: clinical experience from three cases
Source: Front Pediatr. 2026 Jul 28;14:1846557. doi: 10.3389/fped.2026.1846557 (PMC13457645; doi:10.3389/fped.2026.1846557)
Supplement: Supplementary file 1 [file Table1.docx]

Table S1 Longitudinal changes in clinical, laboratory, and disease activity parameters before and after telitacicept treatment.

| Subject |  | Case 1 | Case 2 | Case 3 |
| --- | --- | --- | --- | --- |
| Joint symptoms | | | | |
| Number of joints with active arthritis | Pre-telitacicept | 5 | 2 | 2 |
|  | Post-telitacicept | 6 (1m), 3 (2m),  2 (3m), 3 (6m),  1 (9m), 2 (12m) | 1 (2m), 0 (3m),  2 (6m), 0 (9m) | 2 (1m), 0 (2m),  1 (3m) |
| Number of joints with limited range of motion | Pre-telitacicept | 1 | 2 | 3 |
|  | Post-telitacicept | 1 (1m), 1 (2m),  1 (3m), 1 (6m),  1 (9m), 1 (12m) | 1 (2m), 0 (3m),  0 (6m), 0 (9m) | 3 (1m), 1 (2m),  3 (3m) |
| Inflammatory markers | | | | |
| C-reactive protein  (＜8mg/L) | Pre-telitacicept | 29.83 | 59 | 73.87 |
|  | Post-telitacicept | 22.67 (1m), 17.30 (2m), 1.94 (3m), 1.20 (6m), 2.33 (9m), 3.39 (12m) | 55.70 (2m), 2.72 (3m), 0.80 (6m), 6.50 (9m) | 52.17 (1m), 34.32 (2m), 58.00 (3m) |
| Erythrocyte sedimentation rate (0-15mm/h) | Pre-telitacicept | 93.00 | 85.00 | 59.00 |
|  | Post-telitacicept | 85.00(1m), 63.80 (2m), 74.00(3m), 20.00 (6m), 21.00 (9m), 11.00 (12m) | 43.00 (2m), 6.00 (3m), 8.00 (6m), 8.00 (9m) | 20.00 (2m), 73.00 (3m) |
| Immunoglobulin levels | | | | |
| IgG (g/L)  (5.28-21.90 g/L) | Pre-telitacicept | 14.1 | 12.8 | 17.4 |
|  | Post-telitacicept | 7.84 | NA | 13.7 |
| IgA (g/L)  (0.41-2.97 g/L) | Pre-telitacicept | 3.24 | 4.99 | 1.69 |
|  | Post-telitacicept | 0.42 | NA | 0.98 |
| IgM (g/L)  (0.48-2.26 g/L) | Pre-telitacicept | 3.42 | 2.68 | 1.37 |
|  | Post-telitacicept | 0.59 | NA | 0.49 |
| IgE (IU/ml)  (0-165.00 IU/ml) | Pre-telitacicept | 10.20 | 68.40 | 108.00 |
|  | Post-telitacicept | ＜5.00 | NA | 87.20 |
| Assessment | | | | |
| Physician global assessment of disease activity | Pre-telitacicept | 8 | 5 | 4 |
|  | Post-telitacicept | 7 (1m), 4 (2m),  4 (3m), 2 (6m),  2 (9m), 2 (12m) | 3 (2m), 1 (3m),  2 (6m), 1 (9m) | 4 (1m), 2 (2m),  4 (3m) |
| Parent/patient assessment of overall wellbeing | Pre-telitacicept | 6 | 3 | 5 |
|  | Post-telitacicept | 6 (1m), 3 (2m),  3 (3m), 2 (6m),  2 (9m), 1 (12m) | 2 (2m), 1 (3m),  1 (6m), 1 (9m) | 3 (1m), 2 (2m),  3 (3m) |
| Childhood Health Assessment Questionnaire - Disability Index | Pre-telitacicept | 1.25 | 0.13 | 0.25 |
|  | Post-telitacicept | 0.25 (3m), 0.00 (6m), 0.00 (9m), 0.00 (12m) | 0.00 (2m), 0.00 (3m), 0.00 (6m), 0.00 (9m) | 0.13 (1m), 0.00 (2m), 0.13 (3m) |
| Juvenile Arthritis Disease Activity Score 27 joints | Pre-telitacicept | 26.30 | 16.50 | 14.90 |
|  | Post-telitacicept | 25.50 (1m), 14.38 (2m), 14.40 (3m), 7.00 (6m), 5.10 (9m), 5.00 (12m) | 8.30 (2m), 2.00 (3m), 5.00 (6m), 2.00 (9m) | 4.00 (2m), 13.3 (3m) |
